# An MRI Study on Effects of Math Education on Brain Development Using Multi-Instance Contrastive Learning

**DOI:** 10.3389/fpsyg.2021.765754

**Published:** 2021-11-24

**Authors:** Yupei Zhang, Shuhui Liu, Xuequn Shang

**Affiliations:** ^1^School of Computer Science, Northwestern Polytechnical University, Xi'an, China; ^2^Key Laboratory of Big Data Storage and Management, Ministry of Industry and Information Technology, Xi'an, China

**Keywords:** educational cognitive, MRI, mathematical learning, multi-instance learning, contrastive learning, brain development

## Abstract

This paper explores whether mathematical education has effects on brain development from the perspective of brain MRIs. While biochemical changes in the left middle front gyrus region of the brain have been investigated, we proposed to classify students by using MRIs from the intraparietal sulcus (IPS) region that was left untouched in the previous study. On the cropped IPS regions, the proposed model developed popular contrastive learning (CL) to solve the problem of multi-instance representation learning. The resulted data representations were then fed into a linear neural network to identify whether students were in the math group or the non-math group. Experiments were conducted on 123 adolescent students, including 72 math students and 51 non-math students. The proposed model achieved an accuracy of 90.24 % for student classification, gaining more than 5% improvements compared to the classical CL frame. Our study provides not only a multi-instance extension to CL and but also an MRI insight into the impact of mathematical studying on brain development.

## 1. Introduction

Mathematical learning has significant impacts on the brain's plasticity and cognitive functions and has been associated with many quality-of-life and development indices (Beddington et al., 2008; Zacharopoulos et al., 2021). The understanding of these associations could help in utilizing mathematical learning to benefit the individual's development (Baglama et al., 2017; Steffe, 2017; Zacharopoulos et al., 2021). Toward a better understanding of education behaviors, many researchers made a great number of efforts and yielded a wide range of education discoveries and educational tools from psychological measurements to artificial intelligence (AI) techniques (Steffe, 2017; Barzagar Nazari and Ebersbach, 2018; Mammarella et al., 2018; Zhang et al., 2020a, 2021a; Peng et al., 2021a,b).

This paper reviewed related works for Educational Information Science and Engineering (EISE) from the four aspects, i.e., psychological measurement (Mammarella et al., 2018), biological analysis (Zacharopoulos et al., 2021), educational computer engineering (Robertson and Howells, 2008), and educational data science (Zhang et al., 2020a, 2021a). The psychological measurement aims to quantify education behaviors and understand the learning process from sociality and mentality by using statistical and cognitive models, e.g., item response theory (IRT) (Zhang et al., 2019, 2020a). Leslie reviewed the studies from 1901 to the present and augmented that the mathematics curricula should be constructed following children's psychology (Steffe, 2017). Yupei et al. developed the classical psychological IRT model by seeking latent factors in response records to predict student responses to exam questions (Zhang et al., 2019). Robert et al. explored the nature of the relations among prior information to show the effectiveness of the social cognitive theory (Lent et al., 1993). While psychology explores learning behaviors from phenotypes, biological analysis is used extract the intrinsic impact of education on individuals from brain structure or genotypes (Liu et al., 2021; Peng et al., 2021c; Zacharopoulos et al., 2021). By investigating the numerical cognition in the brain, Korbinian et al. determined that numerical cognition is subserved by a frontoparietal network that connects the cortex, basal ganglia, and thalamus (Moeller et al., 2015). Annie et al. explored the association between neural changes and behaviors, suggesting teachers could help students remedy student misconceptions (Brookman-Byrne and Dumontheil, 2020). Brain et al. reviewed specific learning disabilities to understand the complex etiology and co-occurrences, and accordingly underpin the optimization of learning contexts for individual learners (Butterworth and Kovas, 2013). Based on the understanding of learning behaviors, computer engineering is introduced to create automatic tools or intelligent games to aid student learning and instructor teaching (Ng and Chan, 2019; Alur et al., 2020). Oi-Lam et al. examined students mathematics learning with computer-aided learning software and found that the students used 3D CAD to develop spatial skills and to achieve mathematics learning far beyond using formulate and performing procedures (Ng and Chan, 2019). Christos et al. showed mobile game-based learning could further assist students in higher education toward advancing their knowledge level (Troussas et al., 2020). Alberto built a multi-view early warning system with genetic-programming classification rules and the multi-view learning strategy to enhance the prediction (Cano and Leonard, 2019). In this era of big data, educational data science creates a new path toward educational understanding and increasingly becomes a hopeful prospect for education revolution (Bienkowski et al., 2012). With a sparsity learning model (Zhang and Liu, 2020), Yupei et al. proposed a meta-knowledge dictionary learning model that learnt the latent meta-knowledge instead of the traditional manual Q-matrix (Zhang et al., 2020a). They also used the technique of matrix factorization, integrating the side information of students and courses to predict the learning performance on the next-term course (Zhang et al., 2020c). Through assessing the relations between controlling and autonomy-supportive teaching behaviors on 672 students, Nuria et al. showed that controlling teaching behaviors are negatively associated with psychological needs satisfaction and positively associated with procrastination (Codina et al., 2018). More works in educational data science can be referred to in Cristobal's recent review (Romero and Ventura, 2020). Nevertheless, data science needs to consider a wider range of data types in education research.

In recent years, the impact of mathematical learning on brain development has attracted great attention, where the neuroimage is the usually adopted technique (Kershner, 2020; Zacharopoulos et al., 2021). Mariano et al. discussed four specific cases in which neuroscience synergizes with other disciplines to serve education, ranging from very general physiological aspects of human learning to brain architectures, showing that the neuroscience method, tools, and theoretical frameworks have broadened our understanding of the mind in a way that is highly relevant to educational practices (Sigman et al., 2014). Marie et al. used quantitative meta-analyses of fMRI studies to identify brain regions concordant among studies on number and calculation, yielding a topographical brain atlas of arithmetic (Arsalidou and Taylor, 2011). Ching-Lin et al. reviewed the MRI neuroimaging approach in education studies and kinds of learning themes investigated in MRI research and provided objective and empirical evidence to connect learning processes outcomes and brain mechanisms (Wu et al., 2021). Karin et al. used fMRIs to observe brain activation in mathematical calculation, revealing similar parietal and prefrontal activation patterns in children with developmental dyscalculia compared to controls for various conditions (Kucian et al., 2006). To probe the impact of a lack of mathematical education on brain development, Georege et al. took more than 120 fMRIs from adolescent students that were allowed to stop studying math in the United Kingdom (Zacharopoulos et al., 2021). By examining the neurotransmitter concentrations in the brain, they found that the γ-aminobutyric acid (GABA) concentration in the middle frontal gyrus (MFG) is closely associated with mathematical learning and mathematical reasoning. This is evidence that the lack of math education has effects on brain plasticity and cognitive functions.

However, few studies investigated the effects of education on brain development from the perspective of structural neuroimages. The medical image is a technique of probing the intrinsic structure of the human body that is often utilized in disease diagnosis and therapy (Zhang et al., 2020b, 2021b). While the GABA in the MFG was investigated (Zacharopoulos et al., 2021), we in this paper looked into the math-learning impact on brain development from the intraparietal sulcus (IPS) region that is also frequently reported in neuroimaging studies of arithmetic. This study made an attempt to assess the problem of whether math students and non-math students could be separated by using brain MRIs. The used method first cropped the voxel of interest (VOI), i.e., IPS, from the MRI and then fed all VOI image patches to our proposed multi-instance contrastive learning (MiCL) model, followed by a linear classifier for student identification. Our contributions could be summarized in two aspects: (1) We developed the classical CL model into the setting of multi-instance learning to solve our problem formulation. (2) This study aimed to explore the impact of mathematical education from structural brain MRIs.

## 2. Materials and Methods

This study aims to identify math and non-math students by using MRI data to understand the impact of math learning on brain structure in the IPS region. With this purpose, we designed the following workflow: (1) acquiring MRIs from adolescent students including math students and non-math students and cropping all images into the IPS region (Zacharopoulos et al., 2021), (2) designing a classification tool by using CL for image representations and a linear classifier (Chen et al., 2020; Xu et al., 2021), and (3) evaluating the performance and experiment analyses on the student classification.

### 2.1. The Used MRIs

**Figure 1:**
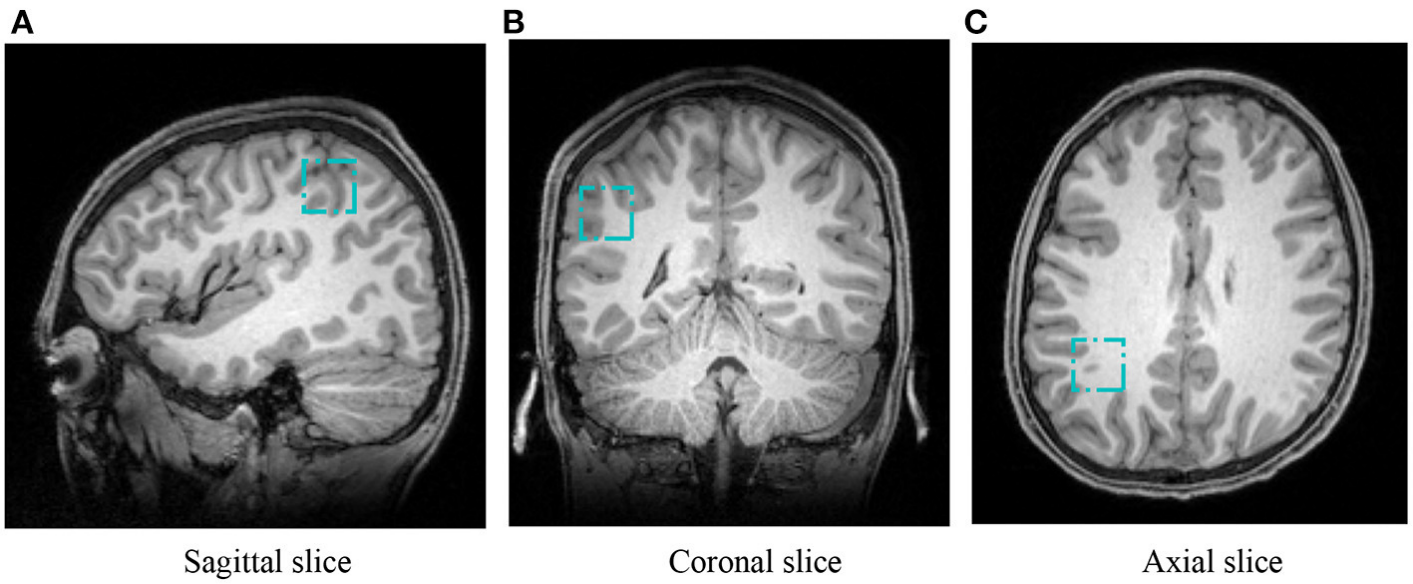
Positions of VOI in a representative T1-weighted MRI for IPS. Three cyan boxes show the IPS from sagittal, coronal, and axial views, respectively. **(A)** Sagittal slice, **(B)** coronal slice, and **(C)** axial slice.

The used MRI data (XNAT Project ID: PN21) were acquired from 16-year-old adolescents that chose to stop or continue math learning in the United Kingdom. Math education was controlled as a single variable to a set math group with 72 students who engaged in A-level math and a non-math group containing 51 students who were not engaged in A-level math. In total, 123 MRIs were acquired on a 3T Siemens MAGNETOM Prisma MRI System equipped with a 32-channel receive-only head coil at the Oxford Centre for Function MRI of the Brain (FMRIB). With an MPRAGE sequence, the anatomical high-resolution T1-weighted MRI was taken by 192 slices, where echo time TE=3.97 ms, repetition time TR =1,900 ms, and voxel size = 1 × 1 × 1 mm. The IPS regions of 20 × 20 × 20 mm were manually defined on the individual's T1-weighted images while the student was lying down in the MR scanner (Zacharopoulos et al., 2021). Acquisition time was 10–15 min per voxel, including planning and shimming. Figure 1 shows the used T1-weighted MRIs together with the left MFG region. We in this study cropped the left IPS region from the T1-weighted MRIs, leading to 3D image VOI patches of 20 × 20 × 20 mm slices. To ensure the computation in deep learning, we normalized all voxels of image patches by

(1) $$\begin{array}{r} I_{ij}=\frac{I_{ij}-x_{min}}{I_{max}-I_{min}} \end{array}$$

where *I*_*ij*_ is an arbitrary pixel in all images; *I*_*max*_ and *I*_*min*_ are the maximal and minimal values among all VOI image voxels, respectively. To train the model in a supervised schema, we shuffled all image slices and took the student's label (i.e., class 1: non-math group, class 0: math group) as slice labels.

### 2.2. Multi-Instance Contrastive Learning

**Figure 2:**
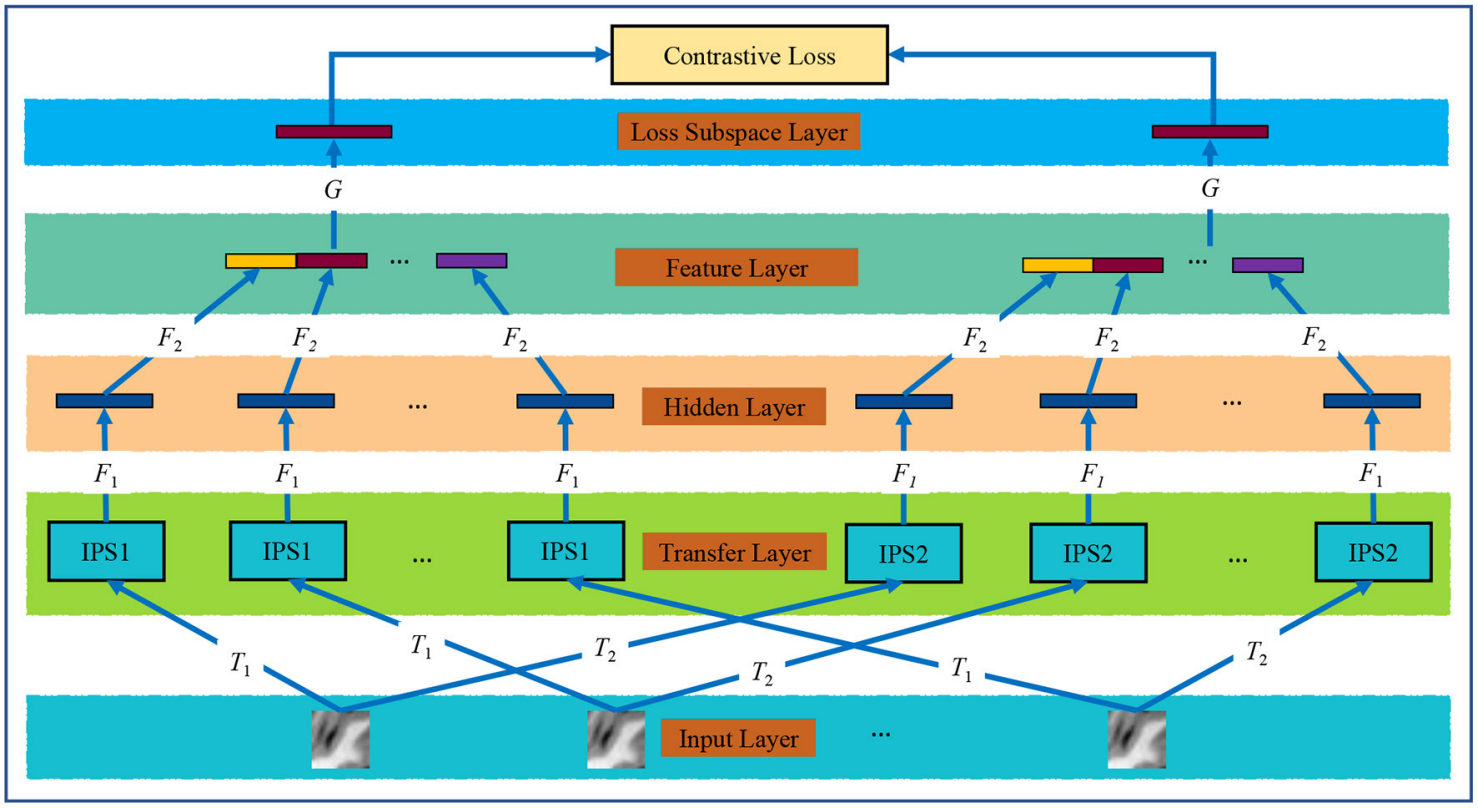
The proposed MiCL model. *T*_1_ and *T*_2_ are two data augmentation operators; *F*_1_ and *F*_2_ are the ResNets; and *G* is a multi-layer perception.

The proposed multi-instance contrastive learning (MiCL) model aims to deal with the problem of student classification where each student involves 20 2D image slices. MiCL includes an input layer of 20 slices per student, a data transform layer for data augmentations, a hidden layer for slice representation learning, a feature layer for student representation learning, and a loss subspace layer for loss computation. Figure 2 shows the framework of the proposed MiCL.

#### 2.2.1. Formulation

Let **X** = {*X*_1_, *X*_2_, ⋯ , *X*_20_} represent student data consisting of 20 instances, where *X*_*i*_ represents an instance for an image slice. All students are denoted by $D={\left\{{\text{X}}_{i},y_{i}\right\}}_{i=1}^{N}$, where *N* is the number of students, and *y*_*i*_ is the label of the *i*-th student. Note that *y*_*i*_ = 1 is for students that have stopped math education, while *y*_*i*_ = 0 is for students that have continued mathematical studying. The problem we will handle in this study is

(2) $$\begin{array}{r} arg\min\overset{N}{\underset{i=1}{\sum }}Q\left(G\left(\text{F}\left({\text{X}}_{i}\right)\right),y_{i}\right) \end{array}$$

where *F* aims to extract the representations from 20 instances per student; G is a classifier that maps **X**_*i*_ to its label *y*_*i*_; and Q is the loss function. In this formulation, the major problem is to learn student representations from all the 20 instances, i.e., the function *F*. A simple method is used to fuse the 20 instances into one student representation, which has been investigated in Dongkuan's work (Xu et al., 2021). While their model is focused on the time series data in a supervised setting, we in this study proposed a new unsupervised model to learn student representations in a multi-instance setting.

#### 2.2.2. Contrastive Learning

Recently, contrastive learning (CL) has become a popular scheme for robust image representation learning and has been widely used in many fields, e.g., text classification (Gao et al., 2021), image classification (Chen et al., 2020), and medical image segmentation (Chaitanya et al., 2020). CL learns the latent image feature by training a nonlinear model on two noisy versions of each data point toward minimizing the difference between them. SimCLR is a representative framework for CL by training a ResNet for image representations and a multiple-layer perceptron (MLP) for loss calculations (Chen et al., 2020). In mathematics, SimCLR is used to seek an optimal solution to the following problem,

(3) $$\begin{array}{r} arg\underset{L,R}{\min}\frac{1}{2N}\overset{N}{\underset{i=1}{\sum }}L\left(R\left(T_{1}\left({\text{X}}_{i}\right)\right),R\left(T_{2}\left({\text{X}}_{i}\right)\right)\right) \end{array}$$

where *T*_1_ and *T*_2_ are the two data augmentation operations from the same family of augmentations; R is the classical ResNet for *F*_1_ and *F*_2_. L is the contrastive loss, which is defined in detail as L = *l*(**z**_*i*_, **z**_*j*_) + *l*(**z**_*j*_, **z**_*i*_), where **z**_*i*_ and **z**_*j*_ are the results from R(*T*_1_(·)) and R(*T*_2_(·)), respectively. The loss function *l*(·) is

(4) $$\begin{array}{r} l\left({\text{z}}_{i},{\text{z}}_{j}\right)=-log\frac{exp\left(sim\left({\text{z}}_{i},{\text{z}}_{j}\right)/\tau \right)}{\sum_{k=1}^{2N}1_{\left[k\neq i\right]}exp\left(sim\left({\text{z}}_{i},{\text{z}}_{k}\right)/\tau \right)} \end{array}$$

where τ is a temperature parameter; **1** is an indicator function; and $sim\left({\text{z}}_{i},{\text{z}}_{j}\right)=\left({\text{z}}_{i}^{T}{\text{z}}_{j}\right)/\left(||{\text{z}}_{i}|{|}_{2}^{2}||{\text{z}}_{j}|{|}_{2}^{2}\right)$.

#### 2.2.3. Objective Function

However, the objective function in Equation (3) fails to handle our multi-instance problem of student classification. To this end, we extended SimCLR into MiCL as

(5) $$\begin{array}{r} arg\underset{L,G,F_{1},F_{2}}{\min}\frac{1}{2N}\overset{N}{\underset{i=1}{\sum }}L\left(G\left({\text{z}}_{1}⊕{\text{z}}_{2}⊕\cdots ⊕{\text{z}}_{20}\right),G\left({\hat{\text{z}}}_{1}⊕{\hat{\text{z}}}_{2}⊕\cdots ⊕{\hat{\text{z}}}_{20}\right)\right) \end{array}$$

where ⊕ is the concentration operation; **z**_*i*_ and ${\hat{\text{z}}}_{i}$ (*i* = 1, ⋯ , 20) are latent representations for the two transformed versions of an input image *X*_*i*_, i.e., **z**_*i*_ = *F*_2_(*F*_1_(*X*_*i*_)). As is shown in Figure 2, we implemented *T*_1_ and *T*_2_ by randomly cropping and resizing, Gaussian blur, translation, and distortions, and *F*_1_ and *F*_2_ by using the classical ResNet, *G* by using MLP, and CL loss by using Equation (4). After all mappings were achieved, we used outputs of the feature layer as student representations for the subsequent classification tasks.

#### 2.2.4. Linear Classifier

To implement the final student classification, this study employs the single-layer neural network that has been investigated in the evaluation of SimCLR (Chen et al., 2020). By denoting **h**_*i*_, the resultant representation for the *i*-th student, the classifier aims to minimize the cost function.

(6) $$\begin{array}{r} L_{0}=\frac{1}{N}\overset{N}{\underset{i=1}{\sum }}-\left[y_{i}log\left(C\left({\text{h}}_{i}\right)\right)+\left(1-y_{i}\right)log\left(1-C\left({\text{h}}_{i}\right)\right)\right] \end{array}$$

where **h** denotes the obtained representation from Equation (5) and C(·) = *Sigmoid*(·) is the activated function mapping student representations to the label space. Equation (6) is the function that measures the binary cross-entropy between the target and the output.

### 2.3. Model Setting and Evaluation

The proposed model shown in Figure 2 was set up in detail as follows. All instances share the same *F*_1_ and *F*_2_, so the two functions are implemented by using the ResNet. The ResNet comprises a convolutional layer with a kernel size of 3 × 3, three residual modules of four bottleneck blocks, and an average pooling layer. The number of channels is 64, 128, 256, 512, 256, 128, and 64, respectively. And the bottleneck block is composed of three convolutional layers with ReLU. Besides, batch normalization (BN) is utilized after each convolutional layer. Our model transfers image instances into a 128-dimensional space, and thus, student features into a 2,560 dimensional space. Then, the MLP for *G* is composed of two fully connected layers of channels 1,024 and 128. Finally, the linear classifier is from 2,560 to 1 and employs the Sigmoid as the activation function to yield the prediction probability. The model was trained by 2,000 iterations with a learning rate of 0.001, and 1,000 iterations trained the linear classifier with a learning rate of 0.005.

In this study, we finally calculated accuracy (ACC), F1-score (F1), and area under the ROC (AUC) on the used 123 MRIs. From the confusion matrix, we calculated the four metrics, i.e., true positive (TP), false positive (FP), false negative (FN), and true negative (TN). ACC and F1 are calculated by

(7) $$\begin{array}{r} ACC=\frac{TP+FN}{TP+FP+TN+FN} \end{array}$$

(8) $$\begin{array}{r} Precision=\frac{TP}{TP+FP} \end{array}$$

(9) $$\begin{array}{r} Recall=\frac{TP}{TP+FN} \end{array}$$

(10) $$\begin{array}{r} F1=2\times \frac{Precision\times Recall}{Precision+Recall} \end{array}$$

and AUC is defined as the area under the ROC. Besides, the two-tailed *t*-test is adopted to compute the *p*-value for the statistic significance test (Zhang et al., 2018). Due to the small-size dataset, we could conduct five-fold cross-validation on the 123 students. That is to say, the model could be trained on four folds and tested on the remaining fold to obtain the average evaluations.

## 3. Result

To have a comparison with SimCLR (Chen et al., 2020), we implemented the student classification by firstly learning an image representation for each slice per student, secondly connecting the 20 representations, and finally reducing them into a 2,560-dimensional PCA subspace (Zhang et al., 2017). In short, we called this method SimCLR through the following context.

### 3.1. Visualization

**Figure 3:**
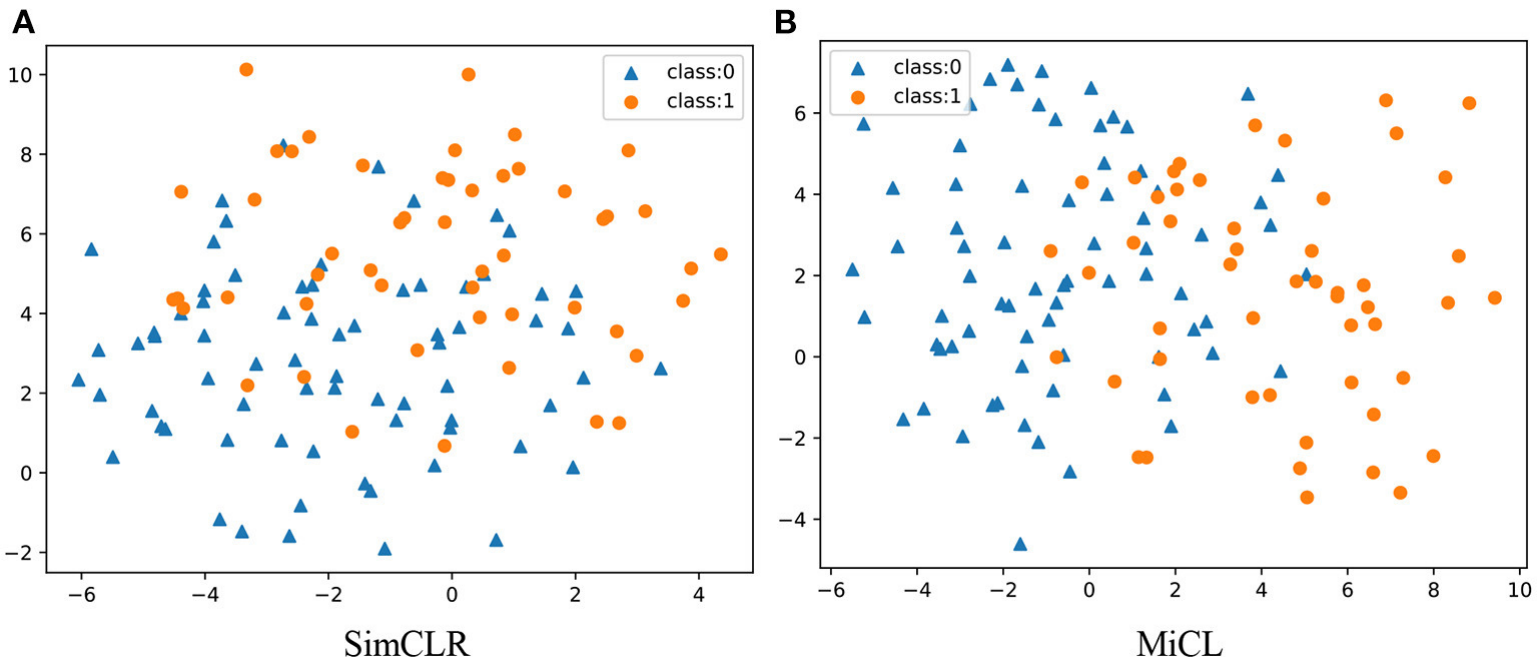
Visualization of the learned representation in 2D subspaces. There are in total 123 students, including 51 students in class 1 and 72 students in class 0. **(A)** SimCLR and **(B)** MiCL.

Figure 3 scatters all 123 student representations from SimCLR and MiCL in the 2D subspace. All obtained representations were reduced into 50-dimensional PCA subspaces and then reduced into 2-dimensional t-SNE subspaces. There were 51 students who stopped math education for class 1 and 72 students who continued math studying for class 0, colored in brown and blue in the figures, respectively. As is shown, the student representations yielded from MiCL could be easily separated between class 1 and class 0, compared to SimCLR, in the 2D t-SNE subspace. This observation potentially suggests that joint learning of the 20 image slices in a multi-instance setting could yield more smart student representations.

### 3.2. Overall Evaluation

**Figure 4:**
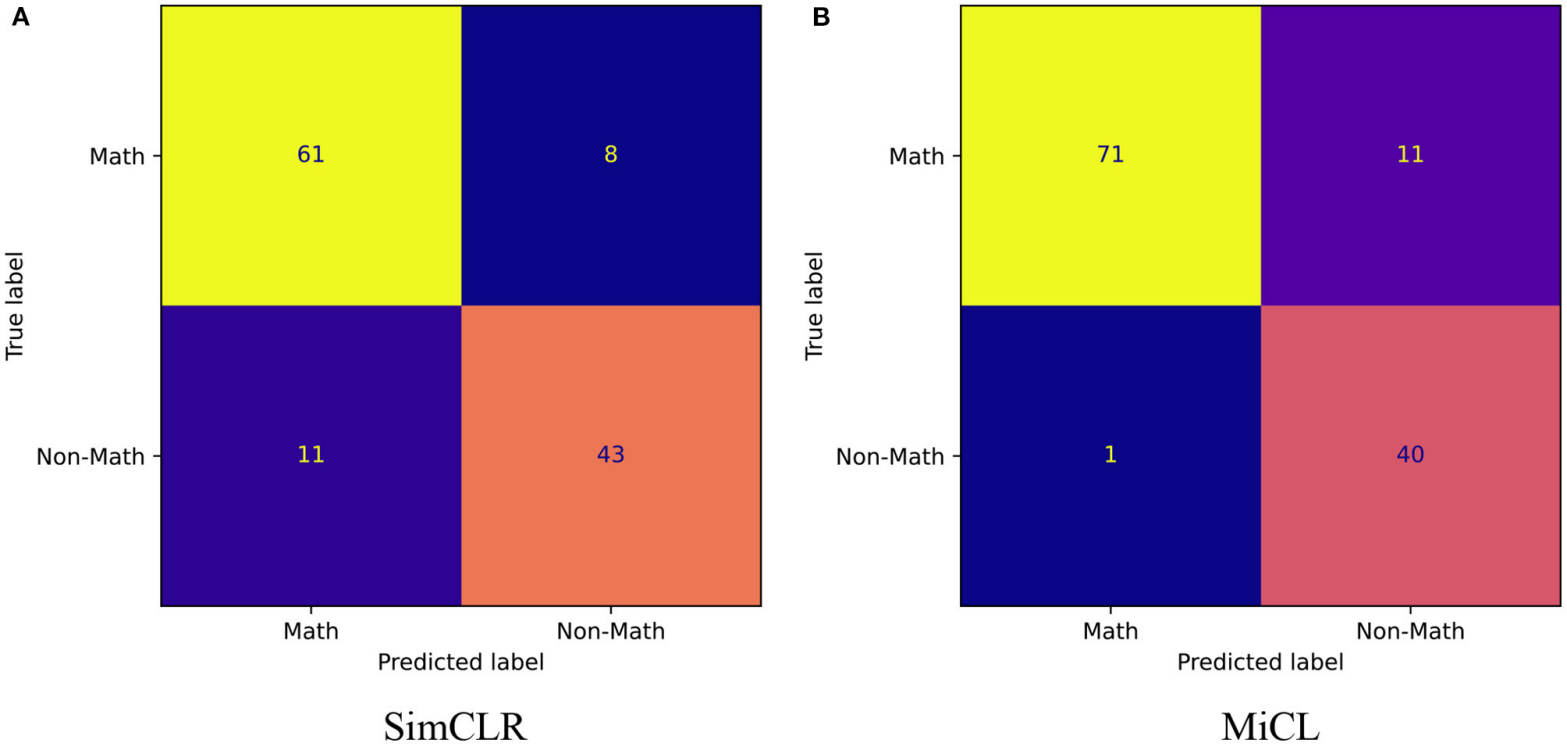
Confusion matrix. The two matrixes show TN, FP, FN, and TP from the classification results of SimCLR and MiCL, respectively. We here considered non-math students as the positive class. **(A)** SimCLR and **(B)** MiCL.

Figure 4 shows the confusion matrixes from SimCLR and the proposed MiCL. Note that this study took the non-math group as the positive class and the math group as the negative class. TP_*SimCLR*_ > TP_*MiCL*_ shows that SimCLR prefers non-math students, while MiCL prefers math students from TN_*MiCL*_ > TN_*SimCLR*_. SimCLR has a big FN while MiCL has a big FP, where FP_*SimCLR*_ = TN_*MiCL*_. That means that SimCLR is better at identifying non-math students, while MiCL is better at identifying math students. However, the proposed MiCL is better overall than SimCLR at classification.

**Figure 5:**
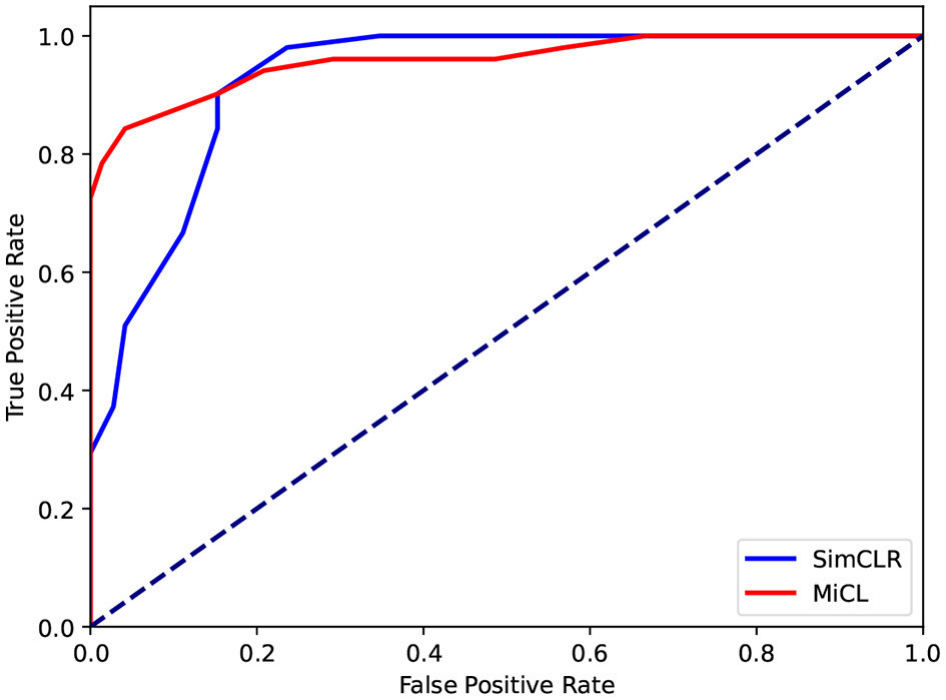
ROCs. The ROCs show the classification performance of SimCLR and MiCL.

Table 1 reports the overall evaluations in terms of the various metrics. Since SimCLR prefers non-math students, SimCLR achieves higher precision than MiCL. But MiCL obtains a higher recall than SimCLR and furthermore results in a higher F1 score. On the other hand, the proposed MiCL gains significant improvements on ACC and AUC by 5 and 3% with *p* < 0.01, respectively. The AUC was obtained by the ROCs, shown in Figure 5. ROCs were plotted by the true positive rate (TPR) against the false positive rate (FPR), showing the classification performance at various thresholds. As is shown, MiCL achieves a higher TPR at a low FPR than SimCLR. Controlling FPR is an important research topic in many fields, e.g., disease diagnosis and drug discovery (Romano et al., 2020). While SimCLR has higher performance at a high FPR, MiCL gains an improvement at AUC that is calculated by the area under ROC in comparison with SimCLR. Overall, the proposed MiCL achieves a better classification performance than SimCLR, while FPR could meanwhile be controlled.

**Table 1:** Evaluation results.

	**ACC**	**Precision**	**Recall**	**F1**	**AUC**
SimCLR	0.8455	0.8431	0.7963	0.8190	0.9289
MiCL	0.9024	0.7843	0.9756	0.8696	0.9578

*Student classification results were calculated for all 123 students in terms of the mentioned metrics, where non-math students were used as positive samples*.

### 3.3. Individual Evaluation

**Figure 6:**
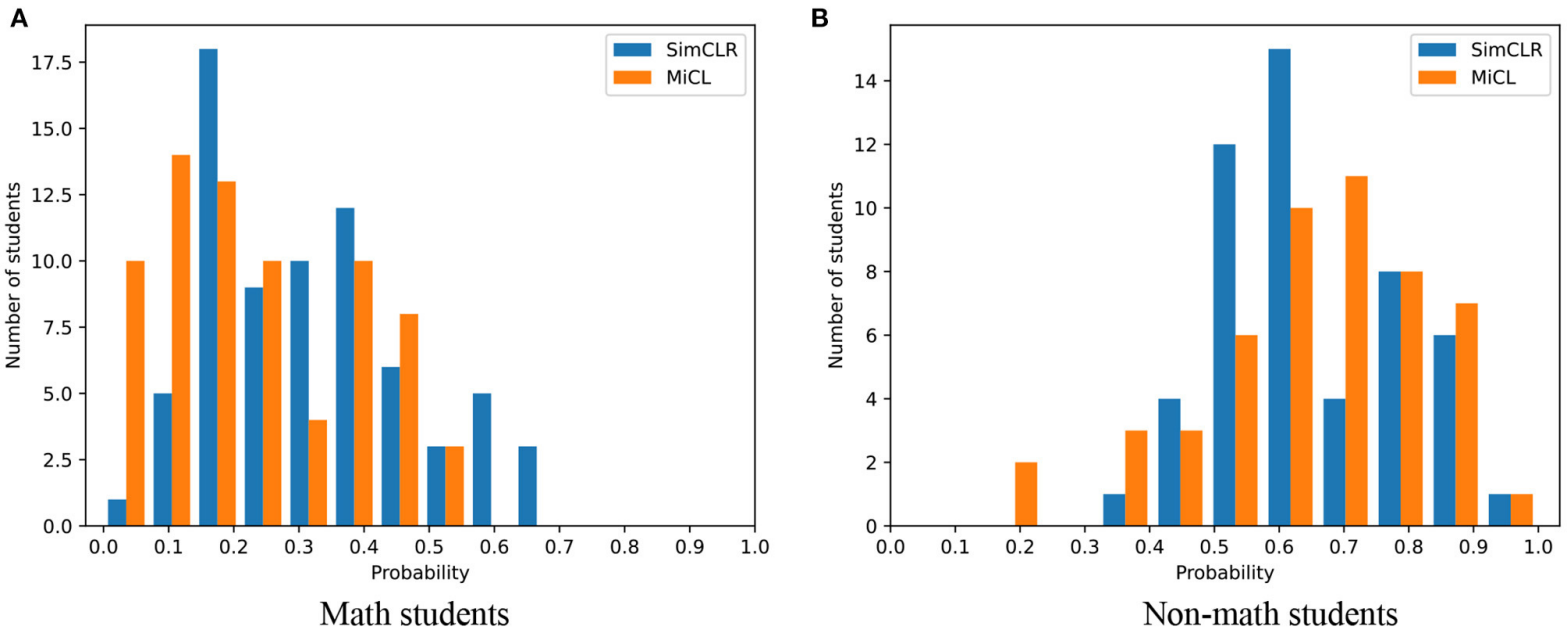
The probability distribution. The distribution of the classification probability for math students and non-math students by SimCLR and MiCL. **(A)** Math students and **(B)** Non-math students.

Figure 6 shows the classification probability for two classes yielded by SimCLR and MiCL. The probability was calculated by normalizing the two outputs to sum 1. That is to say, the sum of the probability belonging to class 1 and the probability belong to class 0 is 100%. In this study, we identified a student to be a math student if the corresponding probability is less than 0.5; otherwise, we identified the student to be a non-math student. As is shown, SimCLR results in most of the probabilities in [0.2, 0.4) for class 0 and most of the probabilities in [0.5, 0.7). And MiCL yields the classification probability concentrated in [0.0, 0.3) for class 0 and the classification probability concentrated in [0.6, 0.9) for class 1. On the other hand, SimCLR leads to more students having a probability of greater than 0.5 for class 0, while MiCL gives rise to more students having a probability of less than 0.5 for class 1. The observation shows that MiCL could yield a more convincing classification for the corrected predictions than SimCLR. Besides, SimCLR leads to more stable predictions for non-math students, and even the probability is concentrated at near 0.5.

Table 2 summarizes the mean and the standard deviation of the classification probability for SimCLR and MiCL, respectively. As is shown, MiCL has a smaller mean with a smaller standard deviation than SimCLR on the tasks of identifying math students. While MiCL has the same mean for non-math students, SimCLR has a smaller standard deviation. However, MiCL yields more confident predictions having benefited from multi-instance joint learning.

**Table 2:** Means and standard deviations.

	**Math student**		**Non-math student**	
	**Mean**	**Standard deviation**	**Mean**	**Standard deviation**
SimCLR	0.3215	0.1581	0.6500	0.1332
MiCL	0.2291	0.1469	0.6501	0.1703

*The results were calculated for all 123 classification probabilities*.

## 4. Conclusion and Discussion

In this paper, we made an attempt to classify students that have stopped studying mathematics and students that have continued their mathematical education by using the popular deep learning technique. To deal with the 3D images, we formulated this problem into multi-instance learning and developed a classical contrastive learning framework in a multi-instance setting.

The proposed MiCL learns the image representation by sharing the weights between the 20 instances and then concatenates 20 image representations, leading to the final student representation. In the two versions of each student, the contrastive loss is employed to encourage a minimal difference. For 123 students, composed of 51 non-math students and 72 math students, MiCL achieves an accuracy of 90.24% that gains a 5% improvement in comparison with SimCLR. Benefitting from the multi-instance joint learning, the same observation has also been obtained for other metrics.

The MRI data have the potential to be used in identifying whether a student has stopped their mathematical education. Both SimCLR and MiCL convey decent accuracy on the classification task of math students or non-math students. Moreover, SimCLR is capable of identifying non-math students more stably, while MiCL prefers to identifying math students. Since the math or non-math student could be separated with a high accuracy using MRIs, mathematical education has a potential impact on adolescent brain development from white matter and gray matter in the IPS region. This conclusion has also been investigated in the work of Karin (Kucian et al., 2006; Zacharopoulos et al., 2021).

There are two points that should be noticed. (1) MiCL gains an insubstantial improvement in accuracy in the 2,560-dimensional subspace in comparison with the 2-dimensional subspace. It may mean that feature selection could be utilized to discover the brain atlas for mathematical studying. (2) Multi-instance joint features maybe contribute more to math-student identification. It potentially means the impact of mathematical studying is more varied on multiple image slices.

Hence, we should uncover the brain atlas that is affected by mathematical education and further discuss the impact on future attainment for adolescents in future works. The attention mechanism could provide more explanations to understand the latent representation, which is our other future consideration (Zhang et al., 2021a). Besides, we will investigate more brain regions that are also related to math learning, e.g., the middle front gyrus (Zacharopoulos et al., 2021), and conduct more experiments to prob the associations between the MRI images and other problems, e.g., student psychology and math anxiety (Barroso et al., 2021).

## Data Availability Statement

Publicly available datasets were analyzed in this study. This data can be found here: http://central.xnat.org.

## Ethics Statement

The studies involving human participants were reviewed and approved by the School of Computer Science at Northwestern Polytechnical University, Xi'an, China. The participants provided their written informed consent to participate in this study.

## Author Contributions

YZ and XS work with the School of Computer Science (SCS) at Northwestern Polytechnical University (NPU), Xi'an, China and funded this study. SL is a Ph.D. student in SCS at NPU, Xi'an, China and collected the data and plotted the figures. YZ designed the study, conducted experiments, and wrote the manuscript. All authors contributed to the article and approved the submitted version.

## Funding

This study was supported in part by National Natural Science Foundation of China (Nos. 61802313 and U1811262), Key Research and Development Program of China (No. 2020AAA0108500), and Reformation Research on Education and Teaching at Northwestern Polytechnical University (No. 2021JGY31).

## Conflict of Interest

The authors declare that the research was conducted in the absence of any commercial or financial relationships that could be construed as a potential conflict of interest.

## Publisher's Note

All claims expressed in this article are solely those of the authors and do not necessarily represent those of their affiliated organizations, or those of the publisher, the editors and the reviewers. Any product that may be evaluated in this article, or claim that may be made by its manufacturer, is not guaranteed or endorsed by the publisher.
